# Supplementary material for: A novel framework for increasing research transparency: Exploring the connection between diversity and innovation
Source: PLoS One. 2025 Jan 9;20(1):e0313826. doi: 10.1371/journal.pone.0313826 (PMC11717280; doi:10.1371/journal.pone.0313826)
Supplement: S1 File — (DOCX) [file pone.0313826.s001.docx]

Table S1. Exploratory and Confirmatory Analysis Steps Required for a Split Sample/Dual Method Protocol

| Exploratory Study | Confirmatory Study |
| --- | --- |
| 1E. 35/65 Random Sample Split. | 1C. Sequester 65% holdout sample until Registered Report published. |
| 2E. Establish passthrough criteria for estimates with respect to statistical significance and magnitude. |  |
| 3E. Frequentist specification testing using 35% sample with each unique specification test documented. |  |
| 4E. Write-up Registered Report that documents exact specifications passed through for *de novo* testing using holdout sample. Document all specifications not passed through in appendix. |  |
| 5E. Publish Registered Report at the Center for Open Science or in public facing working paper archive. Presentation of exploratory findings at academic conferences or departmental seminars encouraged. |  |
|  | 2C. Assess reviews and comments on exploratory results to determine if any changes in *de novo* testing are required prior to any analysis of the holdout sample. If any changes are required, amend Registered Report/working paper. |
|  | 3C. Perform frequentist *de novo* hypothesis tests as stipulated in Registered Report/working paper. Apply false discovery rate and family-wise error rate corrections to test statistics. |
|  | 4C. Use exploratory estimates passed through for testing as weak priors in Bayesian estimation of models. Assess Markov chain Monte Carlo diagnostic tests for convergence and mixing. |
|  | 5C. If any diagnostic tests fail, begin with strategies that do not require any changes to the model such as thinning, increasing the number of iterations, or specifying different MCMC algorithms (e.g., Metropolis-Hasting, Hamilton, Gibbs sampling). If these strategies do not resolve the diagnostic issues, consider re-specifications to address autocorrelation and poor mixing such as mixed models or correlated random effects models. Note any failed diagnostic tests in final output. |
