# Supplementary material for: A novel framework for increasing research transparency: Exploring the connection between diversity and innovation
Source: PLoS One. 2025 Jan 9;20(1):e0313826. doi: 10.1371/journal.pone.0313826 (PMC11717280; doi:10.1371/journal.pone.0313826)
Supplement: S2 File — (DOCX) [file pone.0313826.s002.docx]

Table S2. De Novo Frequentist and Bayesian Estimates of Diversity Indices and Hypothesis Tests, Confirmatory Sample

|  |  | Frequentist | | | | | | | |  | Bayesian | | | | | | |
| --- | --- | --- | --- | --- | --- | --- | --- | --- | --- | --- | --- | --- | --- | --- | --- | --- | --- |
| FDR Order | Ownership Fraction-alization | Log Odds | Std Err | t stat | p-value | FDR α=0.05 Threshold | Odds Ratio | Bon-feronni Lower CI Odds Ratio | Bon-feronni Upper CI Odds Ratio |  | Equal Tailed Lower alpha = 0.000427 | Equal Tailed Upper alpha = 0.000427 | Mean Odds Ratio | Mean Log Odds | Std Dev | Explor-atory Estimate Bayesian Prior |  |
| 1 | MRU^g^ | 1.586 | 0.0953 | 16.64 | <0.0001 | 0.0004273 | 4.887 | 2.989 | 7.989 |  | 4.214 | 5.789 | 4.855 | 1.58 | 0.0517 | 1.588 |  |
| 2 | HMRU^g^ | 1.884 | 0.1135 | 16.6 | <0.0001 | 0.0008547 | 6.58 | 3.665 | 11.81 |  | 5.157 | 10.09 | 6.828 | 1.921 | 0.1043 | 1.955 |  |
| 3 | HMR^g^ | 1.76 | 0.1246 | 14.13 | <0.0001 | 0.001282 | 5.815 | 3.059 | 11.05 |  | 4.957 | 7.532 | 6.013 | 1.794 | 0.0702 | 1.799 |  |
| 4 | EHMRU^ghw^ | 1.72 | 0.1223 | 14.07 | <0.0001 | 0.001709 | 5.587 | 2.973 | 10.49 |  | 4.354 | 7.412 | 5.703 | 1.741 | 0.0949 | 1.978 |  |
| 5 | MU^ghw^ | 1.2 | 0.0858 | 13.99 | <0.0001 | 0.002137 | 3.321 | 2.134 | 5.169 |  | 2.854 | 3.702 | 3.267 | 1.184 | 0.0425 | 1.212 |  |
| 6 | HMU^g^ | 1.571 | 0.1129 | 13.92 | <0.0001 | 0.002564 | 4.814 | 2.689 | 8.616 |  | 3.596 | 6.187 | 4.697 | 1.547 | 0.0875 | 1.653 |  |
| 7 | EMRU^ghw^ | 1.471 | 0.1058 | 13.91 | <0.0001 | 0.002991 | 4.354 | 2.524 | 7.512 |  | 3.542 | 5.25 | 4.297 | 1.458 | 0.0664 | 1.654 |  |
| 8 | EHMR^ghw^ | 1.541 | 0.1113 | 13.84 | <0.0001 | 0.003419 | 4.668 | 2.629 | 8.289 |  | 3.794 | 5.916 | 4.655 | 1.538 | 0.0766 | 1.777 |  |
| 9 | EMR^g^ | 1.228 | 0.0917 | 13.39 | <0.0001 | 0.005385 | 3.415 | 2.128 | 5.48 |  | 2.792 | 4.176 | 3.438 | 1.235 | 0.0629 | 1.375 |  |
| 10 | MR | 1.331 | 0.1019 | 13.06 | <0.0001 | 0.004273 | 3.787 | 2.239 | 6.407 |  | 3.192 | 4.63 | 3.834 | 1.344 | 0.0604 | 1.291 |  |
| 11 | AHMRU^ghw^ | 1.324 | 0.1018 | 13 | <0.0001 | 0.004701 | 3.757 | 2.223 | 6.351 |  | 3.165 | 5.069 | 3.85 | 1.348 | 0.0742 | 1.456 |  |
| 12 | AMRU^ghw^ | 1.118 | 0.0867 | 12.9 | <0.0001 | 0.005128 | 3.058 | 1.956 | 4.781 |  | 2.488 | 3.85 | 3.099 | 1.131 | 0.0748 | 1.191 |  |
| 13 | M | 0.6977 | 0.0567 | 12.31 | <0.0001 | 0.005555 | 2.043 | 1.474 | 2.831 |  | 1.842 | 2.197 | 2.008 | 0.6969 | 0.0253 | 0.7038 |  |
| 14 | AHMR^hw^ | 1.132 | 0.0923 | 12.26 | <0.0001 | 0.005983 | 3.102 | 1.927 | 4.993 |  | 2.464 | 3.81 | 3.155 | 1.149 | 0.0753 | 1.239 |  |
| 15 | EHMU^g^ | 1.428 | 0.1173 | 12.17 | <0.0001 | 0.00641 | 4.169 | 2.277 | 7.631 |  | 3.371 | 5.024 | 4.145 | 1.422 | 0.0648 | 1.674 |  |
| 16 | EMU^g^ | 1.147 | 0.096 | 11.95 | <0.0001 | 0.006838 | 3.148 | 1.919 | 5.163 |  | 2.661 | 3.646 | 3.136 | 1.143 | 0.0509 | 1.312 |  |
| 17 | AMR | 0.8886 | 0.0748 | 11.89 | <0.0001 | 0.007267 | 2.432 | 1.654 | 3.575 |  | 2.034 | 3.009 | 2.447 | 0.8949 | 0.0615 | 0.931 |  |
| 18 | HR^g^ | 0.7957 | 0.0671 | 11.86 | <0.0001 | 0.007692 | 2.216 | 1.568 | 3.132 |  | 1.643 | 2.985 | 2.195 | 0.7864 | 0.1047 | 0.9432 |  |
| 19 | EHM^ghw^ | 1.154 | 0.0974 | 11.86 | <0.0001 | 0.008119 | 3.172 | 1.92 | 5.241 |  | 2.65 | 3.727 | 3.152 | 1.148 | 0.0582 | 1.369 |  |
| 20 | HM^g^ | 1.252 | 0.1063 | 11.78 | <0.0001 | 0.008547 | 3.496 | 2.021 | 6.048 |  | 3.03 | 4.292 | 3.54 | 1.264 | 0.0595 | 1.311 |  |
| 21 | EM^ghw^ | 0.8138 | 0.071 | 11.47 | <0.0001 | 0.008974 | 2.257 | 1.565 | 3.254 |  | 2.006 | 2.551 | 2.232 | 0.803 | 0.0431 | 0.9342 |  |
| 22 | AEHMRU^g^ | 1.273 | 0.111 | 11.46 | <0.0001 | 0.009402 | 3.571 | 2.014 | 6.332 |  | 2.97 | 4.786 | 3.691 | 1.306 | 0.0673 | 1.519 |  |
| 23 | AEHMR^g^ | 1.099 | 0.0969 | 11.35 | <0.0001 | 0.009829 | 3.001 | 1.821 | 4.945 |  | 2.297 | 3.919 | 3.074 | 1.123 | 0.0732 | 1.32 |  |
| 24 | AEMRU | 1.098 | 0.0976 | 11.25 | <0.0001 | 0.01026 | 2.999 | 1.812 | 4.961 |  | 2.337 | 3.833 | 3.019 | 1.105 | 0.0684 | 1.283 |  |
| 25 | AEMR^g^ | 0.9033 | 0.0821 | 11.01 | <0.0001 | 0.01068 | 2.468 | 1.616 | 3.768 |  | 1.982 | 3.073 | 2.518 | 0.9234 | 0.0601 | 1.057 |  |
| 26 | R | 0.6247 | 0.0574 | 10.88 | <0.0001 | 0.01111 | 1.868 | 1.389 | 2.511 |  | 1.537 | 2.254 | 1.899 | 0.6414 | 0.0665 | 0.5619 |  |
| 27 | AHMU^hw^ | 1.069 | 0.1004 | 10.65 | <0.0001 | 0.01154 | 2.912 | 1.736 | 4.886 |  | 2.417 | 3.673 | 2.889 | 1.061 | 0.0645 | 1.204 |  |
| 28 | AMU | 0.846 | 0.0807 | 10.48 | <0.0001 | 0.01196 | 2.33 | 1.537 | 3.533 |  | 2.003 | 2.778 | 2.351 | 0.855 | 0.052 | 0.9195 |  |
| 29 | GHMRU^ghw^ | 1.428 | 0.1384 | 10.32 | <0.0001 | 0.01239 | 4.172 | 2.043 | 8.519 |  | 3.028 | 5.483 | 4.187 | 1.432 | 0.0902 | 1.578 |  |
| 30 | EGHMRU^ghw^ | 1.4 | 0.1389 | 10.08 | <0.0001 | 0.01282 | 4.057 | 1.982 | 8.304 |  | 3.009 | 4.836 | 3.912 | 1.364 | 0.0742 | 1.687 |  |
| 31 | GMRU^ghw^ | 1.224 | 0.1219 | 10.04 | <0.0001 | 0.01325 | 3.4 | 1.813 | 6.376 |  | 2.788 | 4.153 | 3.377 | 1.217 | 0.0645 | 1.302 |  |
| 32 | AEHMU^hw^ | 1.052 | 0.1057 | 9.96 | <0.0001 | 0.01367 | 2.866 | 1.662 | 4.943 |  | 2.306 | 3.455 | 2.881 | 1.058 | 0.0639 | 1.286 |  |
| 33 | EGMRU^hw^ | 1.219 | 0.124 | 9.83 | <0.0001 | 0.0141 | 3.384 | 1.785 | 6.414 |  | 2.952 | 4.08 | 3.501 | 1.253 | 0.0552 | 1.435 |  |
| 34 | AEMU^ghw^ | 0.8702 | 0.0895 | 9.73 | <0.0001 | 0.01453 | 2.387 | 1.505 | 3.787 |  | 2.158 | 2.803 | 2.467 | 0.903 | 0.043 | 1.039 |  |
| 35 | AHM^ghw^ | 0.8183 | 0.0847 | 9.66 | <0.0001 | 0.01496 | 2.267 | 1.465 | 3.508 |  | 1.787 | 2.586 | 2.215 | 0.7953 | 0.0581 | 0.925 |  |
| 36 | AEHM | 0.8471 | 0.0878 | 9.65 | <0.0001 | 0.01538 | 2.333 | 1.484 | 3.668 |  | 1.959 | 2.88 | 2.367 | 0.8616 | 0.0632 | 1.048 |  |
| 37 | AM^g^ | 0.5668 | 0.0607 | 9.34 | <0.0001 | 0.01581 | 1.763 | 1.289 | 2.41 |  | 1.619 | 1.939 | 1.789 | 0.5816 | 0.0292 | 0.6101 |  |
| 38 | AEM | 0.6495 | 0.0696 | 9.33 | <0.0001 | 0.01624 | 1.915 | 1.337 | 2.741 |  | 1.613 | 2.27 | 1.92 | 0.6524 | 0.0512 | 0.7818 |  |
| 39 | EGHMR^hw^ | 1.197 | 0.1283 | 9.32 | <0.0001 | 0.01667 | 3.309 | 1.707 | 6.413 |  | 2.696 | 4.534 | 3.414 | 1.228 | 0.081 | 1.462 |  |
| 40 | AGHMRU^ghw^ | 1.142 | 0.1263 | 9.04 | <0.0001 | 0.01709 | 3.133 | 1.633 | 6.008 |  | 2.459 | 4.43 | 3.34 | 1.206 | 0.0894 | 1.329 |  |
| 41 | AEGHMRU^hw^ | 1.135 | 0.1258 | 9.02 | <0.0001 | 0.01752 | 3.112 | 1.627 | 5.952 |  | 2.404 | 3.765 | 3.133 | 1.142 | 0.0735 | 1.413 |  |
| 42 | EGMR | 0.9907 | 0.1105 | 8.96 | <0.0001 | 0.01795 | 2.693 | 1.523 | 4.762 |  | 2.194 | 3.301 | 2.679 | 0.9856 | 0.0612 | 1.176 |  |
| 43 | EHR^ghw^ | 0.8871 | 0.0997 | 8.9 | <0.0001 | 0.01838 | 2.428 | 1.452 | 4.061 |  | 1.874 | 2.991 | 2.385 | 0.8692 | 0.0892 | 1.234 |  |
| 44 | AGMRU^ghw^ | 0.9876 | 0.1122 | 8.8 | <0.0001 | 0.0188 | 2.685 | 1.505 | 4.789 |  | 2.198 | 3.464 | 2.718 | 0.9998 | 0.0819 | 1.114 |  |
| 45 | AEGMRU^g^ | 0.9979 | 0.1133 | 8.8 | <0.0001 | 0.01923 | 2.712 | 1.512 | 4.866 |  | 2.193 | 3.51 | 2.757 | 1.014 | 0.0734 | 1.217 |  |
| 46 | EGHMU^ghw^ | 1.153 | 0.1312 | 8.79 | <0.0001 | 0.01966 | 3.168 | 1.611 | 6.231 |  | 2.529 | 4.038 | 3.196 | 1.162 | 0.0895 | 1.428 |  |
| 47 | GHMU^ghw^ | 1.143 | 0.1336 | 8.55 | <0.0001 | 0.02008 | 3.136 | 1.574 | 6.244 |  | 2.585 | 4.262 | 3.251 | 1.179 | 0.0707 | 1.301 |  |
| 48 | EGMU | 0.9613 | 0.1127 | 8.53 | <0.0001 | 0.02051 | 2.615 | 1.463 | 4.676 |  | 2.228 | 3.13 | 2.616 | 0.9618 | 0.054 | 1.162 |  |
| 49 | GHMR^g^ | 1.188 | 0.1395 | 8.51 | <0.0001 | 0.02094 | 3.28 | 1.597 | 6.734 |  | 2.665 | 4.55 | 3.408 | 1.226 | 0.0754 | 1.318 |  |
| 50 | HRU^ghw^ | 1.031 | 0.1221 | 8.44 | <0.0001 | 0.02137 | 2.805 | 1.494 | 5.266 |  | 2.167 | 3.191 | 2.632 | 0.9677 | 0.0668 | 1.179 |  |
| 51 | RU^g^ | 0.8569 | 0.1024 | 8.37 | <0.0001 | 0.02179 | 2.356 | 1.389 | 3.995 |  | 1.964 | 2.6 | 2.269 | 0.8194 | 0.046 | 0.889 |  |
| 52 | AEGHMR^g^ | 0.9667 | 0.1163 | 8.31 | <0.0001 | 0.02222 | 2.629 | 1.444 | 4.789 |  | 2.098 | 3.445 | 2.675 | 0.9838 | 0.0775 | 1.222 |  |
| 53 | GMU^g^ | 0.9179 | 0.111 | 8.27 | <0.0001 | 0.02265 | 2.504 | 1.413 | 4.438 |  | 2.151 | 3.192 | 2.577 | 0.9465 | 0.0559 | 1.002 |  |
| 54 | GMR | 0.943 | 0.1174 | 8.04 | <0.0001 | 0.02308 | 2.568 | 1.402 | 4.703 |  | 2.133 | 3.165 | 2.56 | 0.94 | 0.0687 | 0.9932 |  |
| 55 | ER^ghw^ | 0.6484 | 0.0807 | 8.03 | <0.0001 | 0.0235 | 1.912 | 1.261 | 2.9 |  | 1.645 | 2.106 | 1.864 | 0.6227 | 0.0439 | 0.8578 |  |
| 56 | AEGMR | 0.8198 | 0.1021 | 8.03 | <0.0001 | 0.02393 | 2.27 | 1.341 | 3.843 |  | 1.759 | 2.793 | 2.292 | 0.8293 | 0.0621 | 1.011 |  |
| 57 | EHRU^ghw^ | 1.115 | 0.1392 | 8.01 | <0.0001 | 0.02436 | 3.049 | 1.487 | 6.251 |  | 2.366 | 3.782 | 3.016 | 1.104 | 0.0727 | 1.466 |  |
| 58 | EGHM | 0.9091 | 0.1142 | 7.96 | <0.0001 | 0.02479 | 2.482 | 1.378 | 4.473 |  | 1.799 | 3.002 | 2.487 | 0.9109 | 0.0738 | 1.153 |  |
| 59 | AEGHMU^g^ | 0.9437 | 0.1196 | 7.89 | <0.0001 | 0.02521 | 2.57 | 1.386 | 4.762 |  | 2.072 | 3.331 | 2.611 | 0.9597 | 0.0745 | 1.209 |  |
| 60 | AEGMU^g^ | 0.8042 | 0.1048 | 7.67 | <0.0001 | 0.02564 | 2.235 | 1.302 | 3.837 |  | 1.86 | 2.788 | 2.266 | 0.8178 | 0.0636 | 1.008 |  |
| 61 | EGM^hw^ | 0.701 | 0.0919 | 7.63 | <0.0001 | 0.02607 | 2.016 | 1.255 | 3.237 |  | 1.765 | 2.468 | 2.058 | 0.7217 | 0.052 | 0.8621 |  |
| 62 | AGHMR^g^ | 0.9436 | 0.1243 | 7.59 | <0.0001 | 0.02649 | 2.569 | 1.353 | 4.878 |  | 2.048 | 3.215 | 2.602 | 0.9564 | 0.0811 | 1.109 |  |
| 63 | ERU^ghw^ | 0.9192 | 0.1213 | 7.58 | <0.0001 | 0.02692 | 2.507 | 1.341 | 4.688 |  | 1.996 | 2.983 | 2.507 | 0.9192 | 0.0655 | 1.168 |  |
| 64 | AGHMU | 0.9201 | 0.1225 | 7.51 | <0.0001 | 0.02735 | 2.51 | 1.334 | 4.72 |  | 2.027 | 3.219 | 2.579 | 0.9475 | 0.0749 | 1.108 |  |
| 65 | AGMU^hw^ | 0.7614 | 0.1046 | 7.28 | <0.0001 | 0.02778 | 2.141 | 1.248 | 3.672 |  | 1.899 | 2.529 | 2.209 | 0.7925 | 0.0475 | 0.8857 |  |
| 66 | AGMR^ghw^ | 0.7748 | 0.1068 | 7.25 | <0.0001 | 0.0282 | 2.17 | 1.251 | 3.764 |  | 1.795 | 2.612 | 2.161 | 0.7708 | 0.069 | 0.8746 |  |
| 67 | AEGHM^ghw^ | 0.7583 | 0.1066 | 7.11 | <0.0001 | 0.02863 | 2.135 | 1.232 | 3.699 |  | 1.816 | 2.58 | 2.165 | 0.7724 | 0.0597 | 0.9949 |  |
| 68 | AEGM^g^ | 0.6141 | 0.0896 | 6.85 | <0.0001 | 0.02906 | 1.848 | 1.164 | 2.934 |  | 1.577 | 2.219 | 1.859 | 0.6203 | 0.0515 | 0.7848 |  |
| 69 | GHM^ghw^ | 0.8311 | 0.123 | 6.76 | <0.0001 | 0.02949 | 2.296 | 1.217 | 4.33 |  | 1.91 | 2.88 | 2.325 | 0.8436 | 0.0725 | 0.9649 |  |
| 70 | AEHRU^g^ | 0.7999 | 0.1204 | 6.65 | <0.0001 | 0.02991 | 2.225 | 1.196 | 4.139 |  | 1.805 | 3.008 | 2.217 | 0.7963 | 0.079 | 1.098 |  |
| 71 | EH^ghw^ | 0.5227 | 0.0798 | 6.55 | <0.0001 | 0.03034 | 1.687 | 1.118 | 2.545 |  | 1.527 | 2.065 | 1.752 | 0.5606 | 0.0638 | 0.8201 |  |
| 72 | AEHR | 0.6038 | 0.0942 | 6.41 | <0.0001 | 0.03077 | 1.829 | 1.125 | 2.973 |  | 1.548 | 2.386 | 1.874 | 0.628 | 0.0639 | 0.8762 |  |
| 73 | EHU^ghw^ | 0.826 | 0.1298 | 6.37 | <0.0001 | 0.03119 | 2.284 | 1.17 | 4.46 |  | 1.808 | 2.727 | 2.263 | 0.8169 | 0.0688 | 1.163 |  |
| 74 | GM^g^ | 0.5801 | 0.0911 | 6.36 | <0.0001 | 0.03162 | 1.786 | 1.116 | 2.858 |  | 1.592 | 2.055 | 1.795 | 0.5849 | 0.0426 | 0.6345 |  |
| 75 | AERU | 0.6637 | 0.1049 | 6.33 | <0.0001 | 0.03205 | 1.942 | 1.131 | 3.335 |  | 1.575 | 2.475 | 1.943 | 0.6642 | 0.0651 | 0.883 |  |
| 76 | AGHM^g^ | 0.6934 | 0.1142 | 6.07 | 0.0001 | 0.03248 | 2.001 | 1.11 | 3.605 |  | 1.595 | 2.629 | 2.042 | 0.7139 | 0.0762 | 0.855 |  |
| 77 | AHRU | 0.6841 | 0.113 | 6.06 | 0.0001 | 0.0329 | 1.982 | 1.107 | 3.549 |  | 1.561 | 2.609 | 1.99 | 0.6879 | 0.0781 | 0.8716 |  |
| 78 | EGHRU | 0.8089 | 0.134 | 6.04 | 0.0001 | 0.03333 | 2.245 | 1.125 | 4.48 |  | 1.907 | 3.01 | 2.36 | 0.8585 | 0.0813 | 1.168 |  |
| 79 | AER | 0.4623 | 0.0777 | 5.95 | 0.0001 | 0.03376 | 1.588 | 1.064 | 2.37 |  | 1.395 | 1.953 | 1.618 | 0.4812 | 0.0497 | 0.6443 |  |
| 80 | EU^ghw^ | 0.6086 | 0.1034 | 5.89 | 0.0002 | 0.03419 | 1.838 | 1.079 | 3.132 |  | 1.552 | 2.106 | 1.816 | 0.5969 | 0.0582 | 0.8256 |  |
| 81 | AGM^g^ | 0.529 | 0.0915 | 5.78 | 0.0002 | 0.03461 | 1.697 | 1.059 | 2.721 |  | 1.469 | 2.013 | 1.707 | 0.535 | 0.0471 | 0.6231 |  |
| 82 | E | 0.2818 | 0.0489 | 5.77 | 0.0002 | 0.03504 | 1.325 | 1.03 | 1.705 |  | 1.2 | 1.449 | 1.328 | 0.2834 | 0.0353 | 0.4189 |  |
| 83 | ARU^hw^ | 0.5441 | 0.0962 | 5.66 | 0.0002 | 0.03547 | 1.723 | 1.049 | 2.829 |  | 1.365 | 2.151 | 1.728 | 0.5471 | 0.0878 | 0.6467 |  |
| 84 | EGRU^hw^ | 0.6763 | 0.1196 | 5.65 | 0.0002 | 0.03589 | 1.967 | 1.061 | 3.645 |  | 1.725 | 2.4 | 2.041 | 0.7133 | 0.0558 | 0.9438 |  |
| 85 | HU^hw^ | 0.7086 | 0.1308 | 5.42 | 0.0003 | 0.03632 | 2.031 | 1.035 | 3.987 |  | 1.676 | 2.272 | 1.991 | 0.6887 | 0.0477 | 0.9012 |  |
| 86 | AEHU | 0.5947 | 0.11 | 5.4 | 0.0003 | 0.03675 | 1.812 | 1.028 | 3.196 |  | 1.551 | 2.327 | 1.854 | 0.6173 | 0.06 | 0.8721 |  |
| 87 | AEGHRU^g^ | 0.6737 | 0.125 | 5.39 | 0.0003 | 0.03718 | 1.961 | 1.03 | 3.736 |  | 1.538 | 2.616 | 2.004 | 0.6952 | 0.0827 | 1.002 |  |
| 88 | EGHR^ghw^ | 0.5671 | 0.1058 | 5.36 | 0.0003 | 0.03761 | 1.763 | 1.021 | 3.043 |  | 1.338 | 2.149 | 1.709 | 0.5359 | 0.0784 | 0.9043 |  |
| 89 | AEGRU^g^ | 0.5721 | 0.112 | 5.11 | 0.0005 | 0.03803 | 1.772 | 0.994 | 3.158 |  | 1.421 | 2.176 | 1.769 | 0.5706 | 0.0638 | 0.827 |  |
| 90 | AEU^ghw^ | 0.4596 | 0.0901 | 5.1 | 0.0005 | 0.03846 | 1.583 | 0.995 | 2.52 |  | 1.323 | 1.831 | 1.571 | 0.452 | 0.0477 | 0.6523 |  |
| 91 | U | 0.5032 | 0.1029 | 4.89 | 0.0006 | 0.03889 | 1.654 | 0.973 | 2.812 |  | 1.417 | 1.883 | 1.661 | 0.5072 | 0.0435 | 0.5603 |  |
| 92 | AHR^ghw^ | 0.4215 | 0.0862 | 4.89 | 0.0006 | 0.03932 | 1.524 | 0.977 | 2.377 |  | 1.214 | 1.944 | 1.502 | 0.4066 | 0.0757 | 0.589 |  |
| 93 | GHRU | 0.5759 | 0.119 | 4.84 | 0.0007 | 0.03974 | 1.779 | 0.963 | 3.286 |  | 1.249 | 2.322 | 1.771 | 0.5717 | 0.0841 | 0.801 |  |
| 94 | AEH^hw^ | 0.3863 | 0.0802 | 4.82 | 0.0007 | 0.04017 | 1.472 | 0.973 | 2.225 |  | 1.306 | 1.822 | 1.534 | 0.4276 | 0.0577 | 0.6229 |  |
| 95 | EGR^ghw^ | 0.4334 | 0.0901 | 4.81 | 0.0007 | 0.04059 | 1.542 | 0.969 | 2.455 |  | 1.27 | 1.8 | 1.512 | 0.4135 | 0.053 | 0.6613 |  |
| 96 | EGHU^hw^ | 0.5778 | 0.1208 | 4.78 | 0.0007 | 0.04102 | 1.782 | 0.956 | 3.323 |  | 1.419 | 2.229 | 1.796 | 0.5855 | 0.0734 | 0.917 |  |
| 97 | AEGHR^ghw^ | 0.4876 | 0.1068 | 4.57 | 0.001 | 0.04145 | 1.628 | 0.939 | 2.824 |  | 1.351 | 1.999 | 1.617 | 0.4804 | 0.0659 | 0.792 |  |
| 98 | EGU^ghw^ | 0.4482 | 0.1014 | 4.42 | 0.0013 | 0.04188 | 1.566 | 0.928 | 2.641 |  | 1.287 | 1.926 | 1.598 | 0.469 | 0.0613 | 0.6871 |  |
| 99 | AE | 0.2584 | 0.0584 | 4.42 | 0.0013 | 0.04231 | 1.295 | 0.958 | 1.75 |  | 1.183 | 1.496 | 1.309 | 0.2696 | 0.0359 | 0.3997 |  |
| 100 | AEGHU^hw^ | 0.4988 | 0.1156 | 4.31 | 0.0015 | 0.04273 | 1.647 | 0.907 | 2.99 |  | 1.273 | 1.98 | 1.61 | 0.476 | 0.0734 | 0.8081 |  |
| 101 | GRU^ghw^ | 0.4557 | 0.1065 | 4.28 | 0.0016 | 0.04316 | 1.577 | 0.911 | 2.731 |  | 1.209 | 2.014 | 1.552 | 0.4397 | 0.0833 | 0.5768 |  |
| 102 | AEGR | 0.3897 | 0.0925 | 4.21 | 0.0018 | 0.04359 | 1.476 | 0.916 | 2.379 |  | 1.199 | 1.658 | 1.419 | 0.3503 | 0.0494 | 0.6115 |  |
| 103 | AGHRU^g^ | 0.513 | 0.1224 | 4.19 | 0.0019 | 0.04402 | 1.67 | 0.888 | 3.14 |  | 1.336 | 2.285 | 1.739 | 0.5535 | 0.0852 | 0.7609 |  |
| 104 | AHU^ghw^ | 0.4398 | 0.1076 | 4.09 | 0.0022 | 0.04444 | 1.552 | 0.891 | 2.704 |  | 1.264 | 1.936 | 1.532 | 0.4263 | 0.0686 | 0.6308 |  |
| 105 | AEGU^g^ | 0.4028 | 0.0994 | 4.05 | 0.0023 | 0.04487 | 1.496 | 0.896 | 2.498 |  | 1.293 | 1.781 | 1.504 | 0.4079 | 0.047 | 0.6339 |  |
| 106 | AGRU | 0.4162 | 0.109 | 3.82 | 0.0034 | 0.04529 | 1.516 | 0.864 | 2.66 |  | 1.212 | 1.969 | 1.512 | 0.4133 | 0.07 | 0.5811 |  |
| 107 | EGH^ghw^ | 0.3272 | 0.0878 | 3.73 | 0.0039 | 0.04573 | 1.387 | 0.882 | 2.181 |  | 1.147 | 1.62 | 1.375 | 0.3183 | 0.0539 | 0.6217 |  |
| 108 | AU^ghw^ | 0.3057 | 0.0823 | 3.71 | 0.004 | 0.04615 | 1.358 | 0.888 | 2.076 |  | 1.17 | 1.589 | 1.344 | 0.2959 | 0.0491 | 0.4043 |  |
| 109 | AEGH^g^ | 0.3129 | 0.0946 | 3.31 | 0.0079 | 0.04658 | 1.367 | 0.839 | 2.227 |  | 1.135 | 1.604 | 1.359 | 0.3068 | 0.0554 | 0.5859 |  |
| 110 | EG^ghw^ | 0.2148 | 0.0659 | 3.26 | 0.0086 | 0.04701 | 1.24 | 0.883 | 1.741 |  | 1.094 | 1.388 | 1.244 | 0.2186 | 0.0374 | 0.3928 |  |
| 111 | AEG^hw^ | 0.2293 | 0.0764 | 3 | 0.0133 | 0.04743 | 1.258 | 0.848 | 1.865 |  | 1.071 | 1.417 | 1.247 | 0.2211 | 0.0491 | 0.4163 |  |
| 112 | H^hw^ | 0.1803 | 0.0667 | 2.71 | 0.0221 | 0.04786 | 1.198 | 0.849 | 1.689 |  | 0.921 | 1.499 | 1.215 | 0.1945 | 0.0918 | 0.4541 |  |
| 113 | GHU^hw^ | 0.2941 | 0.1085 | 2.71 | 0.0219 | 0.04829 | 1.342 | 0.813 | 2.216 |  | 1.02 | 1.624 | 1.312 | 0.2715 | 0.0809 | 0.5313 |  |
| 114 | AGHU^g^ | 0.3063 | 0.1166 | 2.63 | 0.0253 | 0.04872 | 1.358 | 0.744 | 2.478 |  | 1.031 | 1.69 | 1.314 | 0.2733 | 0.0744 | 0.5522 |  |
| 115 | AGHR | 0.2576 | 0.1117 | 2.31 | 0.0438 | 0.04914 | 1.294 | 0.727 | 2.301 |  | 1.01 | 1.658 | 1.296 | 0.2592 | 0.0798 | 0.4876 |  |
| 116 | AGU^g^ | 0.2234 | 0.0974 | 2.29 | 0.0447 | 0.04957 | 1.25 | 0.757 | 2.066 |  | 1.072 | 1.492 | 1.265 | 0.2347 | 0.0554 | 0.3802 |  |
| 117 | GHR^g^ | 0.2081 | 0.1016 | 2.05 | 0.0678 | 0.05 | 1.231 | 0.729 | 2.08 |  | 0.957 | 1.572 | 1.224 | 0.2022 | 0.0909 | 0.4183 |  |

*Source: 2018 Annual Business Survey 65% Test Sample, project number P-7504866, Disclosure Review Board approval number CBDRB-FY23-0335. FSRDC project number 2681.
Notes: A = Age, E = Educational Level, G = Sex, H = Ethnicity, M = Education Specialization, R = Race, and U = Foreign-born Status. ^g^* i*ndicates posterior distribution failed to pass Geweke test for stationarity, ^hw^ indicates posterior distribution failed to pass Heidelberg-Welch convergence diagnostic. Industry and firm size fixed effects are not shown.*
