# Supplementary material for: A novel framework for increasing research transparency: Exploring the connection between diversity and innovation
Source: PLoS One. 2025 Jan 9;20(1):e0313826. doi: 10.1371/journal.pone.0313826 (PMC11717280; doi:10.1371/journal.pone.0313826)
Supplement: S3 File — (DOCX) [file pone.0313826.s003.docx]

Table S3. Frequentist Specification Tests of Diversity Indices Not Passed through for Hypothesis Testing, Exploratory Sample

| Ownership Fractionalization | Estimate | Standard Error | Odds Ratio |
| --- | --- | --- | --- |
| A | 0.1219 | 0.0141 | 1.13 |
| G | -0.0024 | 0.0163 | 0.998 |
| GU | 0.314 | 0.0273 | 1.369 |
| GR | 0.1976 | 0.0297 | 1.219 |
| GH | 0.133 | 0.0298 | 1.142 |
| AR | 0.3522 | 0.0255 | 1.422 |
| AH | 0.3159 | 0.0261 | 1.371 |
| AG | 0.1327 | 0.021 | 1.142 |
| AGR | 0.3128 | 0.0297 | 1.367 |
| AGH | 0.2731 | 0.0299 | 1.314 |

*Source: 2018 Annual Business Survey 35% Test Sample, project number P-7504866, Disclosure Review Board approval number CBDRB-FY23-0188, CBDRB-FY20-008. FSRDC project number 2681.
Notes: A = Age, G = Sex, H = Ethnicity, R = Race, and U = Foreign-born Status*
