# Supplementary material for: A novel framework for increasing research transparency: Exploring the connection between diversity and innovation
Source: PLoS One. 2025 Jan 9;20(1):e0313826. doi: 10.1371/journal.pone.0313826 (PMC11717280; doi:10.1371/journal.pone.0313826)
Supplement: S4 File — (DOCX) [file pone.0313826.s004.docx]

Table S4. Final and De Novo Bayesian Estimates of Diversity Indices, Confirmatory Sample

|  | Mixed Effects Model | | | | | |  | Fixed Effects Model | | | | | |
| --- | --- | --- | --- | --- | --- | --- | --- | --- | --- | --- | --- | --- | --- |
| Attribute | Exploratory Estimate & Bayesian Prior | Mean Log Odds | Std Dev | ESS | Equal Tailed Lower (alpha = 0.000427) | Equal Tailed Upper (alpha = 0.000427) |  | Equal Tailed Lower (alpha = 0.000427) | Equal Tailed Upper (alpha = 0.000427) | Mean Odds Ratio | Mean Log Odds | Std Dev | Attribute |
| MRU | 1.588 | 1.641 | 0.0615 | 1999 | 4.166 | 6.29 |  | 4.214 | 5.789 | 4.855 | 1.58 | 0.0517 | MRU^g^ |
| HMRU | 1.955 | 1.955 | 0.0755 | 2626 | 5.507 | 9.469 |  | 5.157 | 10.09 | 6.828 | 1.921 | 0.1043 | HMRU^g^ |
| HMR | 1.799 | 1.751 | 0.0728 | 1572 | 4.554 | 7.382 |  | 4.957 | 7.532 | 6.013 | 1.794 | 0.0702 | HMR^g^ |
| EHMRU | 1.978 | 1.839 | 0.0761 | 2048 | 4.983 | 7.901 |  | 4.354 | 7.412 | 5.703 | 1.741 | 0.0949 | EHMRU^ghw^ |
| MU | 1.212 | 1.269 | 0.0483 | 2306 | 3.025 | 4.191 |  | 2.854 | 3.702 | 3.267 | 1.184 | 0.0425 | MU^ghw^ |
| HMU | 1.653 | 1.655 | 0.067 | 2006 | 4.272 | 6.347 |  | 3.596 | 6.187 | 4.697 | 1.547 | 0.0875 | HMU^g^ |
| EMRU | 1.654 | 1.58 | 0.0596 | 1993 | 3.999 | 5.853 |  | 3.542 | 5.25 | 4.297 | 1.458 | 0.0664 | EMRU^ghw^ |
| EHMR | 1.777 | 1.612 | 0.0689 | 1929 | 3.939 | 6.284 |  | 3.794 | 5.916 | 4.655 | 1.538 | 0.0766 | EHMR^ghw^ |
| EMR | 1.375 | 1.287 | 0.0529 | 2073 | 3.074 | 4.375 |  | 2.792 | 4.176 | 3.438 | 1.235 | 0.0629 | EMR^g^ |
| MR | 1.291 | 1.315 | 0.052 | 1569 | 3.164 | 4.499 |  | 3.192 | 4.63 | 3.834 | 1.344 | 0.0604 | MR |
| AHMRU | 1.456 | 1.446 | 0.0713 | 2007 | 3.28 | 5.624 |  | 3.165 | 5.069 | 3.85 | 1.348 | 0.0742 | AHMRU^ghw^ |
| AMRU | 1.191 | 1.224 | 0.0598 | 1924 | 2.841 | 4.221 |  | 2.488 | 3.85 | 3.099 | 1.131 | 0.0748 | AMRU^ghw^ |
| M | 0.7038 | 0.7184 | 0.0306 | 2040 | 1.851 | 2.269 |  | 1.842 | 2.197 | 2.008 | 0.6969 | 0.0253 | M |
| AHMR^g^ | 1.239 | 1.203 | 0.0634 | 2093 | 2.724 | 4.059 |  | 2.464 | 3.81 | 3.155 | 1.149 | 0.0753 | AHMR^hw^ |
| EHMU | 1.674 | 1.554 | 0.0646 | 1712 | 3.684 | 6.347 |  | 3.371 | 5.024 | 4.145 | 1.422 | 0.0648 | EHMU^g^ |
| EMU | 1.312 | 1.255 | 0.0498 | 2556 | 3.034 | 4.149 |  | 2.661 | 3.646 | 3.136 | 1.143 | 0.0509 | EMU^g^ |
| AMR | 0.931 | 0.9483 | 0.0529 | 1379 | 2.192 | 3.089 |  | 2.034 | 3.009 | 2.447 | 0.8949 | 0.0615 | AMR |
| HR | 0.9432 | 0.7814 | 0.0919 | 1125 | 1.549 | 2.959 |  | 1.643 | 2.985 | 2.195 | 0.7864 | 0.1047 | HR^g^ |
| EHM | 1.369 | 1.224 | 0.0536 | 2329 | 2.798 | 4.039 |  | 2.65 | 3.727 | 3.152 | 1.148 | 0.0582 | EHM^ghw^ |
| HM | 1.311 | 1.256 | 0.0546 | 2333 | 2.962 | 4.332 |  | 3.03 | 4.292 | 3.54 | 1.264 | 0.0595 | HM^g^ |
| EM | 0.9342 | 0.8699 | 0.0381 | 1789 | 2.116 | 2.722 |  | 2.006 | 2.551 | 2.232 | 0.803 | 0.0431 | EM^ghw^ |
| AEHMRU | 1.519 | 1.411 | 0.0685 | 2266 | 3.343 | 5.114 |  | 2.97 | 4.786 | 3.691 | 1.306 | 0.0673 | AEHMRU^g^ |
| AEHMR | 1.32 | 1.199 | 0.0598 | 2101 | 2.691 | 4.079 |  | 2.297 | 3.919 | 3.074 | 1.123 | 0.0732 | AEHMR^g^ |
| AEMRU | 1.283 | 1.226 | 0.0572 | 1823 | 2.781 | 4.216 |  | 2.337 | 3.833 | 3.019 | 1.105 | 0.0684 | AEMRU |
| AEMR | 1.057 | 0.988 | 0.0515 | 2165 | 2.305 | 3.139 |  | 1.982 | 3.073 | 2.518 | 0.9234 | 0.0601 | AEMR^g^ |
| R | 0.5619 | 0.5985 | 0.0609 | 2245 | 1.437 | 2.241 |  | 1.537 | 2.254 | 1.899 | 0.6414 | 0.0665 | R |
| AHMU | 1.204 | 1.1867 | 0.0635 | 1636 | 2.616 | 4.059 |  | 2.417 | 3.673 | 2.889 | 1.061 | 0.0645 | AHMU^hw^ |
| AMU | 0.9195 | 0.9482 | 0.0491 | 2053 | 2.149 | 2.965 |  | 2.003 | 2.778 | 2.351 | 0.855 | 0.052 | AMU |
| GHMRU | 1.578 | 1.478 | 0.0816 | 2008 | 3.374 | 5.737 |  | 3.028 | 5.483 | 4.187 | 1.432 | 0.0902 | GHMRU^ghw^ |
| EGHMRU | 1.687 | 1.5 | 0.0776 | 2147 | 3.543 | 5.847 |  | 3.009 | 4.836 | 3.912 | 1.364 | 0.0742 | EGHMRU^ghw^ |
| GMRU | 1.302 | 1.268 | 0.068 | 2101 | 2.642 | 4.402 |  | 2.788 | 4.153 | 3.377 | 1.217 | 0.0645 | GMRU^ghw^ |
| AEHMU | 1.286 | 1.188 | 0.0598 | 2006 | 2.757 | 3.971 |  | 2.306 | 3.455 | 2.881 | 1.058 | 0.0639 | AEHMU^hw^ |
| EGMRU | 1.435 | 1.309 | 0.0649 | 1827 | 2.965 | 4.894 |  | 2.952 | 4.08 | 3.501 | 1.253 | 0.0552 | EGMRU^hw^ |
| AEMU | 1.039 | 0.9855 | 0.0486 | 2471 | 2.302 | 3.093 |  | 2.158 | 2.803 | 2.467 | 0.903 | 0.043 | AEMU^ghw^ |
| AHM | 0.925 | 0.8833 | 0.0515 | 2131 | 2.007 | 2.852 |  | 1.787 | 2.586 | 2.215 | 0.7953 | 0.0581 | AHM^ghw^ |
| AEHM | 1.048 | 0.9373 | 0.0517 | 2362 | 2.168 | 3.053 |  | 1.959 | 2.88 | 2.367 | 0.8616 | 0.0632 | AEHM |
| AM | 0.6101 | 0.6185 | 0.038 | 1891 | 1.625 | 2.101 |  | 1.619 | 1.939 | 1.789 | 0.5816 | 0.0292 | AM^g^ |
| AEM | 0.7818 | 0.7234 | 0.0392 | 2401 | 1.739 | 2.322 |  | 1.613 | 2.27 | 1.92 | 0.6524 | 0.0512 | AEM |
| EGHMR | 1.462 | 1.249 | 0.069 | 1913 | 2.672 | 4.518 |  | 2.696 | 4.534 | 3.414 | 1.228 | 0.081 | EGHMR^hw^ |
| AGHMRU | 1.329 | 1.221 | 0.0744 | 1993 | 2.576 | 4.554 |  | 2.459 | 4.43 | 3.34 | 1.206 | 0.0894 | AGHMRU^ghw^ |
| AEGHMRU | 1.413 | 1.242 | 0.0724 | 1864 | 2.663 | 4.349 |  | 2.404 | 3.765 | 3.133 | 1.142 | 0.0735 | AEGHMRU^hw^ |
| EGMR | 1.176 | 1.034 | 0.057 | 1673 | 2.349 | 3.33 |  | 2.194 | 3.301 | 2.679 | 0.9856 | 0.0612 | EGMR |
| EHR | 1.234 | 1.006 | 0.0693 | 2188 | 2.114 | 3.428 |  | 1.874 | 2.991 | 2.385 | 0.8692 | 0.0892 | EHR^ghw^ |
| AGMRU | 1.114 | 1.061 | 0.0664 | 2163 | 2.329 | 3.586 |  | 2.198 | 3.464 | 2.718 | 0.9998 | 0.0819 | AGMRU^ghw^ |
| AEGMRU | 1.217 | 1.096 | 0.061 | 2402 | 2.375 | 3.796 |  | 2.193 | 3.51 | 2.757 | 1.014 | 0.0734 | AEGMRU^g^ |
| EGHMU | 1.428 | 1.247 | 0.0668 | 2023 | 2.759 | 4.393 |  | 2.529 | 4.038 | 3.196 | 1.162 | 0.0895 | EGHMU^ghw^ |
| GHMU | 1.301 | 1.193 | 0.0702 | 2036 | 2.665 | 4.183 |  | 2.585 | 4.262 | 3.251 | 1.179 | 0.0707 | GHMU^ghw^ |
| EGMU | 1.162 | 1.047 | 0.056 | 2375 | 2.36 | 3.397 |  | 2.228 | 3.13 | 2.616 | 0.9618 | 0.054 | EGMU |
| GHMR | 1.318 | 1.166 | 0.0743 | 2075 | 2.433 | 4.063 |  | 2.665 | 4.55 | 3.408 | 1.226 | 0.0754 | GHMR^g^ |
| HRU | 1.179 | 1.155 | 0.0851 | 2164 | 2.433 | 4.125 |  | 2.167 | 3.191 | 2.632 | 0.9677 | 0.0668 | HRU^ghw^ |
| RU | 0.889 | 0.9652 | 0.0639 | 2239 | 2.088 | 3.27 |  | 1.964 | 2.6 | 2.269 | 0.8194 | 0.046 | RU^g^ |
| AEGHMR | 1.222 | 1.037 | 0.0642 | 2216 | 2.279 | 3.432 |  | 2.098 | 3.445 | 2.675 | 0.9838 | 0.0775 | AEGHMR^g^ |
| GMU | 1.002 | 0.9641 | 0.0574 | 2019 | 2.142 | 3.139 |  | 2.151 | 3.192 | 2.577 | 0.9465 | 0.0559 | GMU^g^ |
| GMR | 0.9932 | 0.9301 | 0.0571 | 2047 | 2.1 | 3.04 |  | 2.133 | 3.165 | 2.56 | 0.94 | 0.0687 | GMR |
| ER | 0.8578 | 0.7494 | 0.0521 | 2078 | 1.794 | 2.457 |  | 1.645 | 2.106 | 1.864 | 0.6227 | 0.0439 | ER^ghw^ |
| AEGMR | 1.011 | 0.88 | 0.0574 | 1737 | 2.004 | 2.929 |  | 1.759 | 2.793 | 2.292 | 0.8293 | 0.0621 | AEGMR |
| EHRU | 1.466 | 1.298 | 0.0769 | 1820 | 2.832 | 4.801 |  | 2.366 | 3.782 | 3.016 | 1.104 | 0.0727 | EHRU^ghw^ |
| EGHM | 1.153 | 0.9536 | 0.0592 | 2014 | 2.111 | 3.199 |  | 1.799 | 3.002 | 2.487 | 0.9109 | 0.0738 | EGHM |
| AEGHMU | 1.209 | 1.045 | 0.0636 | 2220 | 2.29 | 3.615 |  | 2.072 | 3.331 | 2.611 | 0.9597 | 0.0745 | AEGHMU^g^ |
| AEGMU | 1.008 | 0.896 | 0.0541 | 1884 | 2.028 | 2.927 |  | 1.86 | 2.788 | 2.266 | 0.8178 | 0.0636 | AEGMU^g^ |
| EGM^g^ | 0.8621 | 0.7385 | 0.0447 | 2200 | 1.771 | 2.428 |  | 1.765 | 2.468 | 2.058 | 0.7217 | 0.052 | EGM^hw^ |
| AGHMR | 1.109 | 0.9734 | 0.0717 | 2085 | 2.125 | 3.431 |  | 2.048 | 3.215 | 2.602 | 0.9564 | 0.0811 | AGHMR^g^ |
| ERU | 1.168 | 1.085 | 0.06 | 1890 | 2.364 | 3.653 |  | 1.996 | 2.983 | 2.507 | 0.9192 | 0.0655 | ERU^ghw^ |
| AGHMU | 1.108 | 0.997 | 0.0692 | 1855 | 2.188 | 3.36 |  | 2.027 | 3.219 | 2.579 | 0.9475 | 0.0749 | AGHMU |
| AGMU | 0.8857 | 0.8282 | 0.056 | 2047 | 1.92 | 2.77 |  | 1.899 | 2.529 | 2.209 | 0.7925 | 0.0475 | AGMU^hw^ |
| AGMR | 0.8746 | 0.8026 | 0.0589 | 2249 | 1.766 | 2.692 |  | 1.795 | 2.612 | 2.161 | 0.7708 | 0.069 | AGMR^ghw^ |
| AEGHM | 0.9949 | 0.8197 | 0.0567 | 2287 | 1.845 | 2.751 |  | 1.816 | 2.58 | 2.165 | 0.7724 | 0.0597 | AEGHM^ghw^ |
| AEGM | 0.7848 | 0.6646 | 0.0462 | 1926 | 1.673 | 2.301 |  | 1.577 | 2.219 | 1.859 | 0.6203 | 0.0515 | AEGM^g^ |
| GHM | 0.9649 | 0.8184 | 0.0603 | 2177 | 1.868 | 2.826 |  | 1.91 | 2.88 | 2.325 | 0.8436 | 0.0725 | GHM^ghw^ |
| AEHRU | 1.098 | 0.9692 | 0.0691 | 2494 | 2.056 | 3.297 |  | 1.805 | 3.008 | 2.217 | 0.7963 | 0.079 | AEHRU^g^ |
| EH | 0.8201 | 0.6222 | 0.0516 | 2243 | 1.567 | 2.171 |  | 1.527 | 2.065 | 1.752 | 0.5606 | 0.0638 | EH^ghw^ |
| AEHR | 0.8762 | 0.7235 | 0.061 | 1851 | 1.606 | 2.565 |  | 1.548 | 2.386 | 1.874 | 0.628 | 0.0639 | AEHR |
| EHU | 1.163 | 1.003 | 0.0648 | 1962 | 2.24 | 3.37 |  | 1.808 | 2.727 | 2.263 | 0.8169 | 0.0688 | EHU^ghw^ |
| GM | 0.6345 | 0.5738 | 0.0428 | 2093 | 1.533 | 2.05 |  | 1.592 | 2.055 | 1.795 | 0.5849 | 0.0426 | GM^g^ |
| AERU | 0.883 | 0.8131 | 0.0589 | 1693 | 1.869 | 2.776 |  | 1.575 | 2.475 | 1.943 | 0.6642 | 0.0651 | AERU |
| AGHM | 0.855 | 0.7178 | 0.0581 | 2134 | 1.688 | 2.559 |  | 1.595 | 2.629 | 2.042 | 0.7139 | 0.0762 | AGHM^g^ |
| AHRU | 0.8716 | 0.8396 | 0.0741 | 1895 | 1.779 | 2.901 |  | 1.561 | 2.609 | 1.99 | 0.6879 | 0.0781 | AHRU |
| EGHRU | 1.168 | 0.9256 | 0.0781 | 2082 | 1.887 | 3.248 |  | 1.907 | 3.01 | 2.36 | 0.8585 | 0.0813 | EGHRU |
| AER | 0.6443 | 0.5635 | 0.0481 | 1923 | 1.493 | 2.086 |  | 1.395 | 1.953 | 1.618 | 0.4812 | 0.0497 | AER |
| EU | 0.8256 | 0.7561 | 0.0451 | 2561 | 1.824 | 2.466 |  | 1.552 | 2.106 | 1.816 | 0.5969 | 0.0582 | EU^ghw^ |
| AGM | 0.6231 | 0.5538 | 0.0452 | 2162 | 1.459 | 2.021 |  | 1.469 | 2.013 | 1.707 | 0.535 | 0.0471 | AGM^g^ |
| E | 0.4189 | 0.3459 | 0.0278 | 2485 | 1.294 | 1.557 |  | 1.2 | 1.449 | 1.328 | 0.2834 | 0.0353 | E |
| ARU | 0.6467 | 0.6832 | 0.0595 | 2485 | 1.569 | 2.374 |  | 1.365 | 2.151 | 1.728 | 0.5471 | 0.0878 | ARU^hw^ |
| EGRU | 0.9438 | 0.7833 | 0.0654 | 2219 | 1.716 | 2.748 |  | 1.725 | 2.4 | 2.041 | 0.7133 | 0.0558 | EGRU^hw^ |
| HU | 0.9012 | 0.8512 | 0.0731 | 2452 | 1.793 | 3.099 |  | 1.676 | 2.272 | 1.991 | 0.6887 | 0.0477 | HU^hw^ |
| AEHU | 0.8721 | 0.749 | 0.0591 | 2038 | 1.719 | 2.513 |  | 1.551 | 2.327 | 1.854 | 0.6173 | 0.06 | AEHU |
| AEGHRU | 1.002 | 0.7941 | 0.071 | 2394 | 1.74 | 2.731 |  | 1.538 | 2.616 | 2.004 | 0.6952 | 0.0827 | AEGHRU^g^ |
| EGHR | 0.9043 | 0.6156 | 0.0689 | 2232 | 1.461 | 2.36 |  | 1.338 | 2.149 | 1.709 | 0.5359 | 0.0784 | EGHR^ghw^ |
| AEGRU | 0.827 | 0.6831 | 0.0626 | 2103 | 1.629 | 2.485 |  | 1.421 | 2.176 | 1.769 | 0.5706 | 0.0638 | AEGRU^g^ |
| AEU | 0.6523 | 0.5908 | 0.0455 | 2422 | 1.548 | 2.113 |  | 1.323 | 1.831 | 1.571 | 0.452 | 0.0477 | AEU^ghw^ |
| U | 0.5603 | 0.6383 | 0.0469 | 2762 | 1.611 | 2.196 |  | 1.417 | 1.883 | 1.661 | 0.5072 | 0.0435 | U |
| AHR^g^ | 0.589 | 0.5118 | 0.0667 | 2598 | 1.336 | 2.109 |  | 1.214 | 1.944 | 1.502 | 0.4066 | 0.0757 | AHR^ghw^ |
| GHRU | 0.801 | 0.6207 | 0.0843 | 2073 | 1.389 | 2.462 |  | 1.249 | 2.322 | 1.771 | 0.5717 | 0.0841 | GHRU |
| AEH | 0.6229 | 0.4846 | 0.0483 | 2390 | 1.373 | 1.975 |  | 1.306 | 1.822 | 1.534 | 0.4276 | 0.0577 | AEH^hw^ |
| EGR | 0.6613 | 0.4787 | 0.0556 | 2083 | 1.336 | 1.949 |  | 1.27 | 1.8 | 1.512 | 0.4135 | 0.053 | EGR^ghw^ |
| EGHU | 0.917 | 0.679 | 0.067 | 1982 | 1.573 | 2.445 |  | 1.419 | 2.229 | 1.796 | 0.5855 | 0.0734 | EGHU^hw^ |
| AEGHR | 0.792 | 0.5585 | 0.0674 | 1865 | 1.416 | 2.176 |  | 1.351 | 1.999 | 1.617 | 0.4804 | 0.0659 | AEGHR^ghw^ |
| EGU | 0.6871 | 0.5367 | 0.0528 | 2411 | 1.443 | 2.018 |  | 1.287 | 1.926 | 1.598 | 0.469 | 0.0613 | EGU^ghw^ |
| AE | 0.3997 | 0.3309 | 0.0338 | 2196 | 1.25 | 1.555 |  | 1.183 | 1.496 | 1.309 | 0.2696 | 0.0359 | AE |
| AEGHU^g^ | 0.8081 | 0.6059 | 0.0645 | 1715 | 1.52 | 2.248 |  | 1.273 | 1.98 | 1.61 | 0.476 | 0.0734 | AEGHU^hw^ |
| GRU | 0.5768 | 0.5026 | 0.0689 | 2341 | 1.294 | 2.064 |  | 1.209 | 2.014 | 1.552 | 0.4397 | 0.0833 | GRU^ghw^ |
| AEGR | 0.6115 | 0.4538 | 0.0543 | 1692 | 1.294 | 1.866 |  | 1.199 | 1.658 | 1.419 | 0.3503 | 0.0494 | AEGR |
| AGHRU^g^ | 0.7609 | 0.6043 | 0.0788 | 1985 | 1.409 | 2.399 |  | 1.336 | 2.285 | 1.739 | 0.5535 | 0.0852 | AGHRU^g^ |
| AHU | 0.6308 | 0.5799 | 0.0626 | 2432 | 1.439 | 2.223 |  | 1.264 | 1.936 | 1.532 | 0.4263 | 0.0686 | AHU^ghw^ |
| AEGU | 0.6339 | 0.4965 | 0.0532 | 1948 | 1.389 | 1.918 |  | 1.293 | 1.781 | 1.504 | 0.4079 | 0.047 | AEGU^g^ |
| AGRU | 0.5811 | 0.4981 | 0.0655 | 2215 | 1.334 | 2.018 |  | 1.212 | 1.969 | 1.512 | 0.4133 | 0.07 | AGRU |
| EGH | 0.6217 | 0.3603 | 0.0552 | 2229 | 1.201 | 1.781 |  | 1.147 | 1.62 | 1.375 | 0.3183 | 0.0539 | EGH^ghw^ |
| AU | 0.4043 | 0.4209 | 0.0446 | 2185 | 1.298 | 1.785 |  | 1.17 | 1.589 | 1.344 | 0.2959 | 0.0491 | AU^ghw^ |
| AEGH | 0.5859 | 0.3705 | 0.055 | 2260 | 1.212 | 1.726 |  | 1.135 | 1.604 | 1.359 | 0.3068 | 0.0554 | AEGH^g^ |
| EG | 0.3928 | 0.2427 | 0.038 | 2322 | 1.116 | 1.447 |  | 1.094 | 1.388 | 1.244 | 0.2186 | 0.0374 | EG^ghw^ |
| AEG | 0.4163 | 0.2776 | 0.0419 | 2013 | 1.155 | 1.501 |  | 1.071 | 1.417 | 1.247 | 0.2211 | 0.0491 | AEG^hw^ |
| H | 0.4541 | 0.1524 | 0.0757 | 1849 | 0.863 | 1.569 |  | 0.921 | 1.499 | 1.215 | 0.1945 | 0.0918 | H^hw^ |
| GHU | 0.5313 | 0.3242 | 0.073 | 2251 | 1.104 | 1.728 |  | 1.02 | 1.624 | 1.312 | 0.2715 | 0.0809 | GHU^hw^ |
| AGHU | 0.5522 | 0.3791 | 0.0682 | 2221 | 1.185 | 1.809 |  | 1.031 | 1.69 | 1.314 | 0.2733 | 0.0744 | AGHU^g^ |
| AGHR^g^ | 0.4876 | 0.277 | 0.0724 | 2431 | 1.05 | 1.685 |  | 1.01 | 1.658 | 1.296 | 0.2592 | 0.0798 | AGHR |
| AGU | 0.3802 | 0.2879 | 0.0539 | 2450 | 1.114 | 1.607 |  | 1.072 | 1.492 | 1.265 | 0.2347 | 0.0554 | AGU^g^ |
| GHR | 0.4183 | 0.1384 | 0.0817 | 2218 | 0.8859 | 1.5 |  | 0.957 | 1.572 | 1.224 | 0.2022 | 0.0909 | GHR^g^ |

| *Source: 2018 Annual Business Survey 65% Test Sample. The Census Bureau has reviewed this data product to ensure appropriate access, use,* | | | | | | | | | |
| --- | --- | --- | --- | --- | --- | --- | --- | --- | --- |
| *and disclosure avoidance protection of the confidential source data (Project no. 7504866, Disclosure Review Board (DRB)* | | | | | | | |  |  |
| *approval numbers: CBDRB-FY23-0335 and CBDRB-FY24-0126).* |  |  |  |  |  |  |  |  |  |
| *Notes: A = Age, E = Educational Level, G = Sex, H = Ethnicity, M = Education Specialization, R = Race, U = Foreign-Born Status.* | | | | | | | |  |  |
| *g indicates failure to pass Geweke diagnostic test, hw indicates failure to pass Heidelberger-Welch diagnostic test. ESS = Effective Sample Size.* | | | | | | | | | |
