# Supplementary material for: A novel framework for increasing research transparency: Exploring the connection between diversity and innovation
Source: PLoS One. 2025 Jan 9;20(1):e0313826. doi: 10.1371/journal.pone.0313826 (PMC11717280; doi:10.1371/journal.pone.0313826)
Supplement: S6 File — (DOCX) [file pone.0313826.s006.docx]

**Table S5. Representative SAS Code**

| *De Novo* Frequentist Fixed Effects Model  Proc surveylogistic data = holdout_test varmethod=jackknife;  class naics2 firmsz ; /* specify arrays for industry and firm size effects*/  model newtomarket (event = ‘1’) = EHMRU naics2 firmsz / CLOdds alpha=0.0004273;  repweights DAGJK_WGT1-DAGJK_WGT10;  weight TAB_WGT_RDIT;  run; |
| --- |
| *De Novo* Bayesian Fixed Effects Model  Proc qlim data = holdout_test plots=(trace autocorr density) diag=all;  class naics2 firmsz ;  model newtomarket = naics2 firmsz EHMRU / discrete(d=logit);  prior intercept ~ normal(mean=0, var=100); prior EHMRU ~ normal(mean=1.978, var=100);  prior naics2_0 naics2_11 naics2_21 naics2_23 naics2_31 naics2_32 naics2_33 naics2_42 naics2_44 naics2_45 naics2_48 naics2_49 naics2_51 naics2_52 naics2_53 naics2_54 naics2_55 naics2_56 naics2_61 naics2_62 naics2_71 naics2_72 naics2_firmsz_1 firmsz_2 firmsz_3 firmsz_4 firmsz_5 firmsz_6 firmsz_7 firmsz_8 ~ normal(mean=0, var=100);  bayes seed=78324 ntu=100 mintune=20 maxtune=20 nmc=10000 stats(alpha=0.0004273)=summary interval;  weight TAB_WGT_RDIT;  run; |
| Final Bayesian Mixed Effects Model  Proc bglimm data = holdout_test plots=(trace autocorr density) diag=all;  class naics2 firmsz ;  model newtomarket (event = ‘1’) = EHMRU naics2 / distribution=binary;  random intercept / subject = firmsz;  run; |
