# Supplementary material for: A novel framework for increasing research transparency: Exploring the connection between diversity and innovation
Source: PLoS One. 2025 Jan 9;20(1):e0313826. doi: 10.1371/journal.pone.0313826 (PMC11717280; doi:10.1371/journal.pone.0313826)
Supplement: S7 File — (DOCX) [file pone.0313826.s007.docx]

Figure S2. Diagnostic Trace, Autocorrelation, and Posterior Density Plots for EHMRU Coefficient Estimate Using PROC BGLIMM Mixed Effects Model


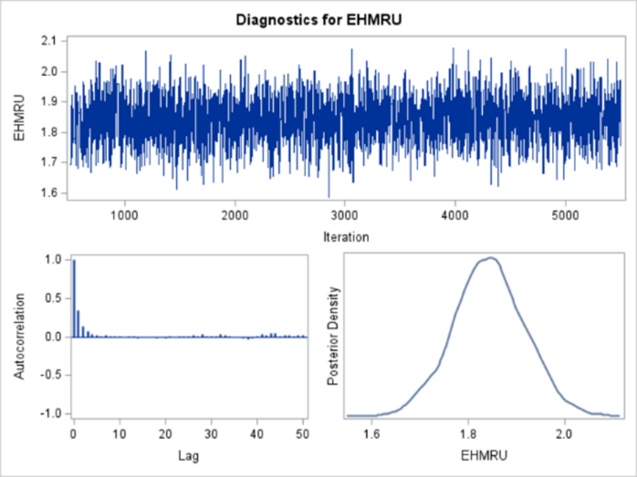


*Source: 2018 Annual Business Survey 65% Test Sample. The Census Bureau has reviewed this data product to ensure appropriate access, use, and disclosure avoidance protection of the confidential source data (Project no. 7504866, Disclosure Review Board (DRB) approval numbers: CBDRB-FY23-0335 and CBDRB-FY24-0126).*
