# Supplementary material for: A novel framework for increasing research transparency: Exploring the connection between diversity and innovation
Source: PLoS One. 2025 Jan 9;20(1):e0313826. doi: 10.1371/journal.pone.0313826 (PMC11717280; doi:10.1371/journal.pone.0313826)
Supplement: Attachment — Submitted filename: Response to Reviewers.docx [file pone.0313826.s008.docx]

Response to Reviewers

Reviewer #1

The statistical framing of this article in the abstract does not seem problematic but it is not new or a contribution. Similarly, the discussion in the introduction about the credibility crisis in applied research is also unrelated to the contribution of this article. Following best practices (or well known practices) is a necessary condition of publication, but not sufficient and not the points to lead with. I would remove both.

One of the contributions of the manuscript is the introduction of Bayesian methods to the split sample protocol for reducing the likelihood of false discovery. Because the split sample protocol imposes a significant statistical power cost on the analysis, Bayesian methods are an effective way to lessen this cost. We have not found other analyses implementing this combined split sample/dual method protocol. If such work exists, we would appreciate citations for such work and will revise our discussion accordingly.

The contribution seems to be about the causal effect of diversity of ownership teams on business innovation. I suggest leading with that.

The method introduced and demonstrated provides an implementable approach to address the credibility crisis in applied research. Accordingly, we believe this is a primary contribution to the article. While the findings concerning ownership diversity and innovation may be regarded as the main research outcome contribution, the main contribution concerning *communication* and *public debate* is related to the methods to reduce false discovery.

The paper claims that “The first step in reducing the set of estimates is to select a single measure that satisfies the requisite axioms discussed below“, but why is a single measure required? Why can’t we study multiple dimensions of this multidimensional concept? Having a single measure is convenient, but what’s the reason for this? If the separate dimensions (your axioms and other features) are correlated, you’ll only learn that if you study the dimension separately, at least at first.

Alternative measures can be proposed and used, but our introduction of the axiomatic approach to diversity measurement makes the selection of our single measure transparent. The argument against multiple measures applied to 7 different diversity dimensions is that the “researcher degrees of freedom” quickly becomes very large. Moreover, the probability of false discovery increases as research degrees of freedom increase. The research approach we are arguing against is the nontransparent investigation of a slew of alternative measures that result in the “preferred” measure being published, accompanied by a post hoc justification. All possible results are estimated and reported by constructing a single measure that satisfies explicit axioms. A single diversity measure addresses the multiple comparison problem by facilitating false discovery rate and family-wise error rate adjustments. There may be conceptual disagreements with the validity of our measure, and we would encourage other researchers to develop and test alternative measures if the same approach to transparency is followed. What critical axioms does our measure miss? Why are our axioms that the new measure does not satisfy invalid? These debates can flourish under an axiomatic approach. However, productive debate is much less efficient if diversity measurement searches are part of unreported specification searches.

If you do have one dimension, and we try to satisfy the (reasonable) axioms proposed, then the authors claim that they have a measure proposed satisfies them. This is merely stated. It seems true, but it needs to be proven. But more importantly, is this the unique measure that satisfies the axioms? A proof that this is unique would be useful. (A somewhat similar example of a uniqueness proof, done by building on entropy, is in doi:10.1093/pan/mpl011). If not, then do the empirical results vary depending on which measure (among those that fit the axioms) is used?

An empirical demonstration of how the diversity index changes with changes in the number of owners, the number of unique groups, and ownership concentration has been added to the manuscript on page 7. A comparison with the commonly used ethnolinguistic fractionalization measure is also provided.

The 2018 *annual* business survey seems like a useful dataset, but why stop in 2018? The article has the laudable training/test framework, and so why not -- following that setup -- use a purely out of sample test set with one of the subsequent years of data?

The main reason for limiting the analysis to a single dataset is that this is the situation that most researchers interested in implementing a split sample/dual method protocol will be in. In addition, the 2018 ABS (reference year 2017) is more than twice as large as the following years (2019-2022), given the Census Bureau’s interest in having more information available in the Economic Census years. Finally, using observations from the same year eliminates one possible explanation for qualitative differences between exploratory and confirmatory tests due to temporal shifts in the phenomenon. All the external factors are the same, so change in the phenomenon over time is not a possible explanation.

A causal identification assumption is required to make Equation 1 useful. This needs to be clearly stated and justified. I’m not sure that is possible here, but it at least needs to be addressed. It is the central point of the entire paper.

No claims concerning causality are made in the manuscript. The analysis is useful because it provides transparent evidence of the association between ownership diversity and innovation. This objective is clarified on page 2, and the limitation is identified in the Discussion on page 17. A causal analysis would be required to support claims that increasing diversity of ownership teams will increase innovation.

Equation 1 also makes big homogeneity assumptions (such as beta1 not varying by the fixed effects). These should be tested and explored. I would suggest that the authors first run their analysis separately within different firm size and industry classes and then only put them together into one equation if the data support that. More likely there are different types of effects in different industries or types of firms, but this will be apparent if you look rather than assume.

Including industry and firm size fixed effects in microdata analysis is a widely accepted procedure for controlling possible confounds of the relationship interest (see Akcigit, U. and Goldschlag, N., 2023. Measuring the characteristics and employment dynamics of U.S. inventors (No. w31086). National Bureau of Economic Research doi:10.3386/w31086, as one of many examples). The homogeneity assumption of differences in the likelihood of reporting innovation being expressed as a shift in the intercept that differs by industry or firm size is not particularly heroic and is well supported by differences in average innovation rates across industry and firm size. The suggested alternative to do numerous subsample analyses decreases statistical power, increases researcher degrees of freedom by increasing the number of separate analyses, and substantially increases the multiple comparison problem. The Bayesian analysis suggests that a mixed model that uses fixed effects for industry and random effects for firm size provides better estimates, passing diagnostic tests that fail in some estimations when only fixed effects are used. The assumption of fixed effects for industry and firm size that poses no problems for the frequentist analysis does run into problems in the Markov chain Monte Carlo analysis that may be due to a poorly specified model.

The “registered report” framework here is hurting the analysis. The authors are not learning from the data and so the readers are not learning from the authors. I would jettison that report and just do a proper model-free statistical exploration.

We view the split sample/dual method protocol as a middle ground between pre-analysis plans where no learning from the data is allowed and model-free statistical exploration that characterizes the data-dependent analysis that predominates in the social sciences. The structure of our protocol only eliminates learning from the data in the confirmatory stage. More specification testing could have been done in the exploratory stage but was limited, given an interest in simplifying the proof of concept. The important dividend of de novo estimation in the confirmatory stage is that the validity of hypothesis test statistics is preserved. Ensuring that the nominal $p$-value reported in publications are true $p$-values is the first step in addressing the credibility crisis in the social sciences.

Reviewer #2

The manuscript demonstrates a robust and technically sound methodology, utilizing both frequentist and Bayesian approaches to enhance the reliability of findings. The use of a split-sample/dual-method protocol is a novel approach that enhances research transparency and addresses issues of reproducibility. The study addresses a highly relevant and contentious topic with significant implications for diversity and innovation policies. However, there are still few comments and suggestions for improvement as follow:

(1) Complexity of Methodological Explanation: The explanation of the dual-method approach and the statistical techniques used, including Bayesian methods and corrections for multiple comparisons, is highly technical and may be challenging for readers without a strong statistical background. Therefore, it is advisable to Simplify the methodological sections by providing a more straightforward summary or an appendix with detailed explanations and step-by-step guides. Including visual aids such as diagrams to illustrate the research design and statistical methods could also be beneficial.

The multiple comparison problem is better illustrated by explicitly defining the p-value and how the 127 different estimates would make conventional hypothesis tests uninformative. This discussion is added on page 7, starting on line 239. One of the primary objectives of the article is to demonstrate the potential benefits of methodological pluralism, so the encouragement to lay out the protocol more simply is appreciated. We have produced a table outlining the distinct steps in both the previous exploratory analysis and the current confirmatory analysis which is now included in Supplementary Materials. We have also expanded the discussion regarding specifying and estimating Bayesian models that are based on frequentist estimation and have provided more references geared to researchers making their first foray into Bayesian analysis. We include SAS code in the Supplemental Materials for interested readers (Table S5).

(2) More Discussion on Practical Implications is needed: The manuscript provides an extensive methodological discussion but lacks depth in discussing the practical implications of the findings for business practices and policy-making, authors are advised to explain further how findings could influence business strategies, diversity policies, and innovation practices.

One of the authors is a government employee, so there are some limits to the discussion about policy implications or actions that might be taken, given the findings. The major limitation of the analysis for informing the practical implications of the diversity/innovation association is that causality is not established. (This was not the objective of the manuscript). We discuss this limitation in the final section on page 17, beginning on line 537. This analysis focused on providing the most transparent and robust estimates of the association as an impetus for research that plausibly controls for endogeneity and is extended to innovation outcomes mediated in markets. These issues are added to the end of the concluding Discussion section beginning on line 539.

(3) Proofreading Needs, a final proofread to catch minor typographical errors and ensure consistency in terminology and formatting would improve the manuscript’s polish.

Revisions from line editing and proof reading are indicated in track changes.

(4) Two Self-Citation were found, While self-citations are acceptable, ensuring they do not dominate the reference list

Because research using the self-reported innovation questions in the ABS is still quite limited, self-citations are sometimes necessary. Material first developed in the registered report has been added to this manuscript to address comments from reviewers requesting elaboration on the ownership diversity measure.

Reviewer #3: Dear authors,
this is a very interesting paper pursuing several ambitious and relevant goals. I absolutely agree with the need for increased research transparency in social sciences/economics and appreciate the authors’ engagement with these issues and the proposed ideas and protocol. However, considering the different aims and contributions made, the paper would benefit from a clearer focus. This includes, on the one hand, engaging a little more with the existing literature on innovative effects of diversity and, on the other hand, ensuring that the proposed protocol is accessible for the intended audience.

Major points
1. I understand that the conceptual and empirical background on diversity effects is not the focus of this paper and is therefore kept short. However, as an application addressing (in the authors’ words) a “contentious” issue, and certainly one attracting both theoretical, empirical and policy attention, I find the engagement with the literature superficial. I broadly agree with the theoretical reasoning for both innovative advantages and hinderances but would suggest to acknowledge the existing literature on diversity effects a bit more explicitly. This applies especially to the section “Diversity Findings Vulnerable to False Discovery” but also to the sections discussing the results. Some suggestions:
o for an overview of the literature: Ozgen, C. (2021). The economics of diversity: Innovation, productivity and the labour market. Journal of Economic Surveys, 35(4), 1168–1216. <https://doi.org/10.1111/joes.12433>
o for a specific application of team diversity to firm innovation: Brixy, U., Brunow, S., & D’Ambrosio, A. (2020). The unlikely encounter: Is ethnic diversity in start-ups associated with innovation? Research Policy, 49(4), 103950. <https://doi.org/10.1016/j.respol.2020.103950>
o as a seminal reference for the existence of diversity effects: Page, S. E. (2007). The difference: How the power of diversity creates better groups, firms, schools, and societies. Princeton Univ. Press.
o for a review of different measures of diversity: : Nijkamp, P., & Poot, J. (2015). Cultural diversity: A matter of measurement. In The Economics of Cultural Diversity (pp. 17–51). Edward Elgar Publishing.

A fuller discussion from the literature of how diversity may promote or hinder innovation has been added on pages 2-3. A reference to Nijkamp & Poot is very helpful for demonstrating the index search problem that might contribute to false discovery.

I have some concerns regarding the fractionalisation measure proposed here. I think it is important to realise that fractionalisation is a pretty standard indicator in research on diversity generally. There are a few variants (mostly different decompositions) but they are often based on Herfindahl-Hirschmann concentration measures. Considering that the aim here is to reduce risks of false discovery, I am not quite sure why you propose your own improvement of established fractionalisation. This is not to say that I disagree with implementing new measures (to the contrary) but wouldn’t it improve comparability to previous results if you actually used the same measures? In contrast, if the intention is to contribute to the literature on measuring diversity, the proposed indicators would need to be theoretically grounded in the literature, the conceptual ideas of diversity and also analysed and documented vis-a-vis its empirical behaviour (e.g., as a first step, also descriptively). Without this, the diversity analysis here seems to be more of an empirical exercise while it could be a very relevant contribution to the literature on diversity effects in its own right. I would suggest that the authors reflect on their intended contribution and whether, given space constraints, a combination of a new methodological approach and a new indicator can be achieved here without short-changing either of these aims.

Relying on an established fractionalization measure would have been preferred if they would satisfy all the requisite axioms without modification. However, since diversity measures are often used to compare entities of very different sizes, they were designed to be invariant to population size. The small group diversity investigated here imposes different requirements, and the team size can significantly affect the number of different perspectives. We now include a direct comparison of values of the ethnolinguistic fractionalization measure and the ownership fractionalization measure for different values for distinct groups, team size, and concentration of ownership to make assessment of the new measure easier.

A justification for each of the axioms now provides a richer conceptual basis for the measure and demonstrates the value of the axiomatic approach for promoting debate on alternative indices.

3. Following on from the previous point, I have some more concrete concerns with the proposed diversity measures.
a) It took me a few reads to understand Axiom 3. I would suggest that you re-write it for clarity. If I understand correctly, you are referring to the issue that team size affects the possible amount of diversity, i.e. it is harder for a small team to be diverse than for a large one.

The justification added for the team size axiom makes this clearer. Not only does the likelihood of diversity increase with firm size, but larger teams with the same ethnolinguistic fractionalization measure as a smaller team would interact more with other groups’ viewpoints.

b) If I understand your Axiom 3 correctly, then the fact that fractionalisation is “invariant to team (population) size by design” is an issue that is acknowledged in studies using fractionalisation or other indices (see e.g. Brixy et al., 2020). It is usually addressed by explicitly controlling for team size. Why is this not an option (or not sufficient) in your case here?

We do not view team size as a confounding factor that needs to be controlled but rather as intrinsic to diversity in small groups that should reflect the number of diverse nodes in a network. Each diverse node presents the possibility of cognitive conflict that may contribute directly to innovation.

c) As far as I can see, the proposed OF index here only differs from fractionalisation because it raises the shares to the power of n (unique groups) rather than just to the power of 2, as in a HHI. Perhaps I am misunderstanding this, but it is not clear to me how this change addresses Axiom 3, since team size is not reflected in the OF equation, only number of groups. A fully diverse team of three people (i.e. three different groups) is still going to have a lower OF than a fully diverse team of four people. Could you please explain how your modification improves upon the issue of team size and how it compares to the established fractionalisation indices?

We have added tables comparing the value of the E.F. and the OF measures.

d) The notion of a composite diversity indicator is very interesting and could open interesting avenues from a conceptual perspective. However, here, almost no details on the construction of this indicator is provided, and its theoretical foundations, advantages or drawbacks are not discussed. Also, isn’t the COF just an average across fractionalisation indices of different dimensions? What does this imply for composite diversity as a concept?

On page 7, we explicitly identify the COF used in the current analysis as an unweighted (or equally weighted) measure of diversity across the included dimensions and identify the possibility of using different weights for different policy focuses.

4. A table showing the considered levels of the various diversity dimensions would be helpful (i.e. how many educational specialisations do you consider?) both for interpretation and also for transparency reasons (since different aggregation levels for the groups could affect the results). Also, the number and categories of control fixed effects (i.e. industry and firm size controls) would be helpful.

Table 1, describing the categorical variables used in the OF in detail, is now provided on page 5. The firm size and industry categories are explicitly identified in the text.

5. I understand that you cannot identify family-owned businesses in the data. But since this characteristic is emphasised as an explanation throughout the text, I am wondering if the authors could provide a sense of the proportion of family-owned businesses. How common are family-owned businesses in the U.S. and in which industries? Can the authors provide any evidence supporting the claim that family-owned businesses are more likely to show higher degrees of diversity in age or gender than other types of businesses? And finally, since the authors explain the negative effects of gender and age diversity by referring to their relatively high prevalence among family-owned businesses, is it also established that these family-owned businesses are less innovative? In principle, a negative effect of gender or age diversity could be theorised: if innovation is supported by the combination of different and novel experiences, perhaps the experiences among different age groups or genders are not distinct enough to provide innovative impulses? This would of course need to be investigated further and the imprecision in the estimates remains an issue but I am wondering whether this perhaps deserves a little more detail in interpretation.

A respecification, including a control for family-owned businesses, is technically possible. However, this option has been strongly discouraged by the Federal Statistical Research Data Center administrator as it would be unlikely to be cleared by the Disclosure Review Board. The DRB is reluctant to clear minor modifications to a specification as these small differences between models may pose a disclosure threat. That is why the suggestion in the manuscript is to include these controls in future analyses. We have provided references to support our claim that family-owned businesses are less likely to report innovation on page 9. However, this issue has also raised a possible limitation in applying a fully transparent split sample protocol to confidential data with tightly controlled access, as found in the FSRDCs. Without the disclosure concerns, the registered report would be expected to reproduce all specification tests used to arrive at the models pass through for confirmation. This may not be possible within the FSRDC due to the concerns raised by releasing findings from similar specifications. We address this limitation in the Discussion section and propose how this issue might be addressed by providing the number of alternative specifications tested even if the parameter estimates from each unique specification cannot be published.

6. Depending on the intended audience, you may want to provide more detail on how the Bayesian method is implemented. The authors write (p.5) “What we used from the exploratory stage and how we arrived at reasonable values for the missing input should assist those with little experience with Bayesian estimation.” and I see that some effort is made in that respect, but I still feel that the details of the procedures could be clarified (e.g. here some (probably) trivial questions showing my ignorance, but why are the priors set with standard deviation 10? What does the traceplot look like here? Why do you use the Geweke and Heidelberger-Welch test and not the other ones?). The results are mostly clearer to follow but if this paper is indeed intended to show a protocol that can be applied rather easily, I would consider some more details on how to do that.

Thank you for the suggestion. We added the following to support the standard deviation prior of 10:

“The priors used to estimate the model parameters in Equation 1 are weakly informative. The priors for the intercept, β_0 is the normal distribution centered on zero with a standard deviation of 10. **The standard deviation of 10 corresponds with a prior variance of 100. Setting the variance to this value means the prior distribution is centered over the parameter value from the exploratory findings but with very wide, symmetric tails. Bayesian procedures use simulation methods to generate posterior distribution conditional on prior information. The wide tails associated with a standard deviation of 10 ensure a more extensive search space around the prior learned from the exploratory step, allowing the sampling procedure to explore the posterior distribution more fully.**”

We also include the rationale for using the convergence diagnostics mentioned above:

“Diagnostic statistics providing tests of stationarity and convergence include Raftery-Lewis, Gelman and Rubin, Geweke (Geweke, 1992), and Heidelberger-Welch (Heidelberger and Welch, 1981). The latter two are used in this analysis. **They provide information concerning the Markov chain’s first (mean) and second (variance) moments. The Geweke procedure tests if the mean estimates of a chain have converged (i.e., they are stationary) by comparing the means from the early and later segments of the posterior sample. Using a different statistical procedure, the Heidelberger-Welch (H.W.) tests whether the covariance of the posterior distribution is stationary**.”

We also include trace and correlation plots comparing the proc qlim and proc bglimm results for one of the COF variables (EHRMU) failing both diagnostic test in the *de novo* analysis in the Supplemental Materials (Figures S1 and S2).

We also include representative SAS code in the Supplemental Materials for interested readers (Table S5). Due to disclosure concerns, we are not able to provide the complete SAS code.

7. Since PLOS ONE addresses an international audience, the country of analysis should be stated clearly at least once. At the moment, the article speaks of “the Census Bureau’s Federal Statistical Research Data Center” (p. 2) and “the 2018 Annual Business Survey” but never actually specifies that we are talking about the U.S. context here. Also, please consider whether more information on the used survey may be required (e.g. what is the coverage or sampling of this survey?) for readers unfamiliar with this dataset.

More information on the Annual Business Survey and its role in the U.S. statistical system is provided on page 2, beginning on line 78. We also mention in the Abstract that the United States if the country of analysis.

1. p.8: “These results, provided in Supplementary Materials, are qualitatively very similar to the de novo confirmatory estimates but represent final estimates of the effect of team diversity on innovation.” This sentence is not clear to me – what does it mean when you say that those are “final” estimates?

“…but represent final estimates of the effect of team diversity on innovation as the failures to pass diagnostics test in the de novo estimation are resolved.”

2. There are a few strong statements that would need to be qualified or supported with sources:
o p. 4 “Finally, previous research finds that diversity and homophily are positively associated with innovation….” needs a reference.

Added.

o p. 3 “A composite ownership fractionalization measure, which combines two or more diversity measures, represents how diversity is experienced”- I am doubtful that any quantitative measure could ever capture the actual experience of diversity.

“represents how diversity is experienced” changed to “provides an empirical analogue to the multidimensional character of diversity experienced in everyday life.”

3. In the notes for Table 1 and Table 2, the category M is missing, i.e. the only one not defined (by exclusion it seems to be education specialisation?).

Thank you for catching this. Corrected.

4. p.8. When referring to tables 1 and 2 in the text, it would be helpful to remind the reader what the condition of inclusion in these tables was, i.e. that they show the most and least precise estimates.

See revisions on page 13 (line 413, 454 and 457).
