# Supplementary material for: A novel framework for increasing research transparency: Exploring the connection between diversity and innovation
Source: PLoS One. 2025 Jan 9;20(1):e0313826. doi: 10.1371/journal.pone.0313826 (PMC11717280; doi:10.1371/journal.pone.0313826)
Supplement: Attachment — Submitted filename: Response to reviewers.docx [file pone.0313826.s009.docx]

**Thank you for your valuable submission.**

1) Please double-check grammar (e.g. punctuation and verb tense);

*In track changes.*

2) Please double-check refs (e.g. abbreviations, page numbers, and sentence case need to be corrected);

*Checked. Page dashes made consistent with* [ICMJE](http://www.nlm.nih.gov/bsd/uniform_requirements.html). *Sentence case for titles with colons now conforms with “Vancouver” style, as outlined in th*e [ICMJE](http://www.nlm.nih.gov/bsd/uniform_requirements.html).

3) Although the title is not concerning, I believe the authors could improve by focusing on a specific aspect of the innovation. For example, Bayesian estimation is correct, but for a broad audience, it won't be as strong as "novel framework" or even "Robust methods for...". These are only simple examples requesting authors to make it more 'punchy' yet important and clear for researchers too;

*Thanks for the suggestion. “A novel framework for increasing research transparency: exploring the connection between diversity and innovation” is punchier and avoids the contentious topic redundancy that should be clear from the subject matter.*

4) Consider refining the Abstract, particularly in terms of language and the presentation of information (e.g. "Bayesian estimation replaces traditional [...] in the confirmatory stage" seems a little bit more direct and strong statement). The authors are strongly encouraged to make the Abstract more engaging, emphasising the novelty and relevance of the research;

- Complex terms such as 'priors' and 'posteriors' could perhaps be introduced in the Introduction rather than the Abstract to make the latter more accessible;
- If the options for Title are not particularly engaging, consider rephrasing terms like 'Bayesian methods' to make it engaging for both experts and the broad audience. This also applies to the wording in the Abstract;
- Please work on a strong introductory line that emphasises the relevance of the study;
- Provide consistent results based on your study with its parameters;
- Additionally, strengthen the Abstract reinforcing the importance of the findings, and make the practical implications of the study more explicit;

*We see the main contribution of this paper as being methodological: demonstrating a framework that can directly address the credibility crisis in applied research. Accordingly, we retain the original first sentence of the abstract. We have removed more technical terms from the abstract and streamlined the description of the protocol. The magnitude of the impact of diversity is now included in the abstract but we are reticent to outline practical implications of the study as are findings are correlational rather than causal.*

5) Simplify sections where the focus shifts towards stats and use clearer language to explain complex concepts;

*We have examined these sections and believe that they provide readers using statistical techniques in their own research adequate information to understand what was done.*

6) The Introduction needs to provide a clearer explanation of how the chosen diversity measure aligns with the stated axioms and why it was selected over alternatives;

*The introduction now discusses the axiomatic selection of a diversity measure to reduce researcher degrees of freedom. A fuller discussion of how the ownership fractionalization measure satisfies these axioms has been added to the second section on “Diversity findings vulnerable to false discovery.”*

7) The absence of a Methods section or, at the very least, an in-depth explanation of search, data interpretation and presentation focussing on stats is a little bit worrying. I'd highly encourage the authors to reshape the sections preceding the Tables, focusing on explaining how the data was gathered and how it will be displayed in terms of stats parameters. A sup section could also be important;

*The discussion in the section “Specification of the innovation and ownership diversity regression equation” provides the justification and interpretation of the variables used in the analysis that are all provided in the Annual Business Survey. An explanation of the statistics to be provided in the confirmatory results tables is provided in the “Exploratory results” section that defines the FWER and FDR corrections to be used for the confirmatory results.*

8) The presentation of OF, EF, and COF is commendable, though the text could benefit from further refinement. Specifically, it could elaborate on: a) how the indices interact with the different dimensions; and b) a clearer and more direct argument (with data from both simulations and practical examples) on how Bayesian methods can help mitigate power loss in frequentist models;

*In response to comments from Reviewer 3 we discuss more fully the logic behind the 4 axioms and how the OF/COF satisfies them. This has allowed us to simplify the EF/OF comparison tables that are now limited to maximal values for different values of the number of groups and number of owners. The demonstration of ownership concentration in the previous tables was ambiguous and has been removed. We also estimated the correlation of the EF and OF indices which is very high (>0.90) for dimension with 2 groups, weakening somewhat as the number of groups in a dimension increase. Results from using the EF index in place of the OF index are qualitatively similar but of smaller magnitude and this is mentioned in the manuscript on page 22.*

- The introduction of the OF and COF indices is innovative, but it would benefit from a more explicit explanation of how they compare to traditional diversity indices beyond the tables. For instance, explaining why the exploratory stage mitigates concerns about subjective priors could help readers;

*The expanded discussion of how the OF satisfies the four axioms should make the measure selection more persuasive to readers. In addition, we have examined the sensitivity of our findings to replacing OF with EF and the results are qualitatively similar, with the two measures being strongly correlated. The main difference between OF and EF is the sensitivity to team size which we argue is essential in the study of small teams where interaction among members is guaranteed. This discussion begins on line 177.*

9) Consider tidying the Tables;

*Many of the statistics presented in Tables 4 and 5 may appear superfluous relative to reporting as commonly done in journal articles, particularly with respect to FDR, FWER (Bonferroni correction) and Bayesian credible intervals. However, all of this information is essential for fully understanding the implications of the multiple comparison problem and assessing the possible advantages of methodological pluralism in this proof-of-concept application. Subsequent papers using the split sample/dual method protocol will be able to present tidier tables only including the Bayesian estimates while referencing this paper to establish advantages with respect to statistical power.*

10) Overall, the Tables are dense with numerous parameters. The abbreviations are unclear without constant reference to the footnotes;

*Abbreviations have been added after the variable name labels in Table 6 that should aid readers scanning the document who may not be looking at table footnotes. Naming COF variables with the diversity dimension labels becomes intractable when the number of diversity dimensions exceeds 3 or 4.*

- Not all readers require both Frequentist and Bayesian estimates to be presented simultaneously. Streamlining the Tables prioritising the most significant data or presenting detailed Bayesian diagnostics separately would enhance clarity;

*Demonstrating the advantages to methodological pluralism is a central objective of the manuscript. Simultaneous presentation of the frequentist and Bayesian estimates accomplishes this. In addition to the demonstration of improved power in the Bayesian estimation, side-by-side estimates may allay concerns from readers unfamiliar with Bayesian methods that the weakly informative prior does not “put a thumb on the scale” of the Bayesian estimate. While this may be a concern for much smaller samples, the side-by-side comparison demonstrates that likelihood dominates with large samples.*

- For the Frequentist model, providing R-squared or adjusted R-squared values would help evaluate how well the model fits the data;

*R-squared in large micro datasets with parsimonious models of complex processes like innovation often provides little basis for evaluation of model fit. It is not included given the large number of statistics in the tables.*

- It is unclear at a glance which estimates are meaningful, given the numerous values and parameters presented;

*The Bonferroni confidence intervals and equal-tailed credible intervals are now bold in Table 4 and 5 as these are the estimates that are most meaningful.*
- While it may be implicit, the use of Forest Plots could be particularly useful in simplifying the density and presentation of the data;

*The objective of presenting a large number of diversity estimates is not to rank or compare them necessarily but rather to provide assurances to the reader that the results have not been cherry-picked. A visual display of credible intervals could reinforce a ranking objective that is not the intent of the manuscript.*

- The same for presentation of priors - or mention before the Tables;

*Inclusion of the priors on Tables 4 and 5 serves several purposes: 1) it makes clear that the prior is an input in the Bayesian estimation; 2) it provides a link back to the exploratory analysis used in the split sample/dual method protocol; and 3) it demonstrates to readers that weakly informed priors in estimations using large datasets have little effect on the final Bayesian estimates other than shrinking the standard deviation in the posterior distribution.*

11) The authors are encouraged to use more references while debating and presenting the arguments in Discussion;

- It'd be valuable to include more refs and explanations on the prevalence of HARKing, claims regarding irreproducible studies, and in-depth references for specific information, such as in line 526: "climate change, public health, diversity";

*References supporting the Discussion section have been expanded considerably. In addition, references cited earlier in the paper are added where appropriate to demonstrate how the current research addresses the problems of data dependent analysis.*

- What 'Registered Report'?;

*[27] from the References now included in the text.*

- Are other studies or meta-analyses relating to diversity and innovation? If so, they should be mentioned and explored;

*We have been unable to find any studies or meta-analyses of ownership diversity and innovation. There are studies of diversity in the workplace but there are very few data sources like the Annual Business Survey that combine an ownership survey (the former Survey of Business Owners) and an innovation survey (the innovation module from the former Business R&D and Innovation Survey). Among OECD countries that follow the Oslo Manual for innovation surveys, the U.S. is the only country that includes information on company owners.*

In addition, consider updating refs, including those by Nosek, Baltagi, or Vehtari, for example. Also, strengthen your argument by citing relevant literature, and provide a clearer summary of the changes made to the ms.

*Reference to Nosek added along with other references in Discussion section that reinforce our argument.*

Wishing you success with the study.

==============================

**Reviewer #3:** Dear Authors,

Thank you for the opportunity to read the revised version of this manuscript. I believe that the paper has much improved and I appreciate the work that the authors have invested. In particularly, the justification for the analysis and the detail of the method description are both much clearer now.

However, I still have concerns with the proposed measure of ownership fractionalisation (OF). While the justification for the axioms is clearer now, there is still no actual engagement with the conceptual meaning of this indicator or even proof that it satisfies the axioms. The “simple modifications” suggested to ensure that the axioms are satisfied are not conceptually explained or justified and seem to be derived purely mathematically, whereas the EF does have a conceptual grounding. This leads to a strange comparison of a well-known and conceptually valuable index with an almost arbitrary-seeming mathematical construct.

The authors instead provide two tables that are supposed to compare the OF with the established fractionalisation indicator based on an HHI. I see two issues with these tables:

1. An easy to fix but important problem is that the rows and columns in table 2 and table 3 seem to be mislabeled (or flipped). It says that the rows are number of owners and columns are unique groups but this does not correspond to the content of the tables. As it is presented now, the table should show OFs for two owners (row 1) across two, three and four unique groups, which would mean that owners can be in more than one group simultaneously. While this is of course possible theoretically, it falls outside the proposed OF indicator (and is rather an example of the COF or other multidimensional measures). In fact, when tracing the numbers in table 2, it is clear that the numbers in the last column (4-equally distributed) correspond to 1 – 4*((1/4)^2) = 0.75, 1 – 4*((1/4)^3) = 0.9375 and 1- 4*((1/4)^4)=0.9844. According to equation 1, the exponent n identifies the number of groups, not the number of owners as the table now suggests. It seems that the labels have been flipped in the tables without adjusting the numbers.

*The original Tables 2 and 3 have been replaced with maximal value tables (justification below) with the corrected row and column labelling.*

2. I also do not agree with the calculations in table 3. Clearly, the EF does not have a “number of owners” dimension but is only calculated for the number of groups. I understand that this is what you are trying to show by repeating the same value in the table but I think that this is an oversimplification and the values in the table are actually not entirely correct or not clearly presented.

Because the EF is calculated based on the relative prevalence of the unique groups (not based on ownership shares) changing the number of owners while keeping the number of groups constant would in fact change the EF value in some cases. Consider the case of 2 unique groups: it is true that the value for two owners and four owners with equal distribution would both be 0.5. But for three owners, the EF would actually be 1-((1/3)^2+(2/3)^2)=0.6667 because the distribution across groups would necessarily be unequal. Since the EF depends on the relative proportions of the groups as determined by both the number of groups and the number of owners, it is actually not entirely “invariant” to team size. The same applies for three unique groups and three versus four owners.

*EF in the literature is used with populations and the resulting population shares across groups, not with small teams with a maximum number of 4. However, applying EF to small teams can result in the anomalies of the measure being sensitive to team size as noted. To address this issue along with the ambiguities related to ownership concentration we have replaced the original tables with maximal value tables. These tables demonstrate the differences in possible range of the indices given changes in the number of groups and number of owners.*
Moreover, the columns using “concentrated” ownership do not really makes sense for the EF since they are not formally captured. Of course, we can just use ownership shares analogously to how we would use population shares here but then the EF-value for some constellations still depends on the distribution across groups. For instance, a team with two unique groups and three owners and concentrated shares could either have an EF of 1 – (0.01^2+0.99^2)=0.0198 (just like a team with two owners) or of 1-(0.02^2+0.98^2)=0.0392. Without more information on the concentration across the two groups, either of these values could occur.

*Ownership concentration has been removed from the EF and OF comparison tables to address the ambiguities of distribution across groups. OF satisfaction of the ownership concentration axiom is now explained in text rather than demonstrating it numerically (line 231 in Manuscript or 241 with Track Changes). OF satisfaction of the other 3 axioms also receives a textual explanation that is demonstrated in the revised Tables 2 and 3.*

In essence: the example comparisons the authors present in table 3 do not actually capture the behaviour of the EF as it is not as static as suggested here but does in fact respond to changes in team size as long as these have an effect on relative prevalence of the groups. Thus, the empirical demonstration that was meant to clarify the behaviour of the OF relative to the EF thus does not help to clarify the added value and/or distinction between the two indices yet but rather highlights the complexity of these measures.

*The EF as used in the literature is not sensitive to population size and this is a valuable property in a measure that is intended to compare diversity across regions or countries of vastly different sizes. When applied to small teams the EF measure can demonstrate some sensitivity to team size as demonstrated. However, the difference between the two is that EF does not represent each owner as a node in a network as OF does. As explained in the text, each owner in a 4-owner team evenly split between two groups would have twice as many interactions with an owner from another group when compared to an evenly split 2-owner team. In contrast, EF is premised on the probability of interacting with a member from another group so the network analogue, and the size of the network, does not apply. For this reason, we believe OF is a better measure for examining diversity in small teams where interaction among members is guaranteed.

EF would be the preferred measure for controlling for diversity in the surrounding population as mentioned in the follow-on research in the final paragraph.*

I would suggest either revising the tables to show the behaviour of the two indices more comprehensively and correctly (perhaps with a less abstract example?) or perhaps also consider other methods of comparison. For instance, I wonder (just out of curiosity) about the simple correlations between the EF and OF measures in your data: Do they tend in the same direction on average? How different are these two indices really?

*Out of an abundance of caution we re-estimated all the equations with the EF (or composite EF) index in place of the corresponding OF or COF index. We have added a discussion that compares EF and OF with respect to correlation on page 13 (Track Changes) and with respect to findings on page 22 (Track Changes). Not surprisingly, EF and OF are very strongly correlated (> 0.90) for the binary dimensions of ethnicity, sex, and foreign-born status and highly correlated for dimensions with more groups. The findings are qualitatively similar using either EF or OF but the magnitudes of OF coefficient estimates are larger, particularly for COF that include dimensions with lots of groups such as Education Specialization or Race.*
